# Incorporating Clinical Trial Data Into Daily Cancer Care

**Published:** 2012-11-01

**Authors:** Jody Pelusi

**Affiliations:** From Arizona Oncology–Sedona, US Oncology, Sedona, Arizona

## Abstract

The objective of clinical trials is to determine the effectiveness and safety of specific interventions. Regulatory agencies, clinicians, and patients depend on clinical trials because they provide the most reliable information about treatment outcomes. The ability to predict how a patient may respond to a given treatment and what potential types, degree, and frequency of adverse events could occur is invaluable. Although data from clinical trials can determine effective treatment options for patients, it is the explanation of what to expect from treatment and how it may affect quality of life that will determine which option a patient chooses. Translating clinical trial data into "real life" can be challenging for the oncology advanced practitioner (AP) because primary and secondary endpoints may differ among clinical trials. This variability can produce confusion when comparing, contrasting, and translating clinical evidence into clinical practice. This article reviews clinical trial endpoints and surrogate markers and describes how findings can influence decision-making and patient care. With social media increasing patients’ awareness and encouraging active participation in their own care, it is imperative that APs be able to articulate clinical trial outcomes along with their strengths, limitations, and life impact.

Despite the fact that metastatic disease remains incurable, individuals with metastatic cancer are living longer with their disease and undergoing multiple lines of therapy. It is important to recognize that clinical trials, as well as hospice/palliative care, are also viable options for patients with metastatic disease, in addition to treatments such as chemotherapy, biotherapy, targeted/novel treatments, and hormonal therapies.

Selecting a treatment should be based on tangible outcomes such as, but not limited to, increase in overall survival (OS) and progression-free survival (PFS). The potential impact of treatments on the quality of life (QOL) of the patient and his or her family members must also be considered, along with overall expectations and life goals.

Oncology advanced practitioners (APs) play a vital role in the management of patients in both the adjuvant as well as metastatic settings because they help guide their patients through the maze of treatment options (Palmieri, Frye, & Mahon, 2009). The decision-making strategy for treatment selection depends not only on national guidelines such as those of the National Comprehensive Cancer Network (NCCN, 2012a; NCCN, 2012b), but also on many other factors. These factors include tumor-related symptoms, previous persistent side effects of treatment, potential future side effects, comorbidities, estimation of survival time, time to expected treatment response, patient preference, performance status, life goals, and overall impact on QOL for both the patient and family (Orlando et al., 2007). The financial and social impact of specific cancer treatments must also be considered when selecting treatment. Thus, patients and caregivers need clear, concise, and patient-centered information about each treatment option in order to truly be able to make an informed decision.

Randomized controlled clinical trials are conducted to determine first-line treatment for metastatic disease. Over the years, various primary and secondary endpoints have been utilized in trials for metastatic disease, including OS, PFS, disease-free survival (DFS), time to tumor progression (TTP), time to treatment failure (TTF), and overall response rate (ORR; Hudis et al., 2007). The selection of clinical trial endpoints has changed over time, secondary to the evolution of clinical trial development, limited trial participation, and increased availability of current treatment options. The variability in endpoints, plus the lack of clarity on which endpoints should be used and when, may create confusion for the AP, especially when trying to compare and contrast study outcomes and translate them to "real life" for patients and families. Moreover, many of the trial designs do not address questions faced by oncologists and APs, such as whether and when to use monotherapy vs. combination therapy and the sequence in which multiple lines of therapies should be administered (Cardoso et al., 2009). This article will review the common endpoints and surrogate markers utilized in clinical trials and describe their impact on treatment decision-making.

## Outcome Measures and Endpoints for Clinical Trials

Before a clinical trial is started, researchers determine which primary and secondary endpoints will be necessary to draw meaningful conclusions about the treatment’s overall effect and its risk/benefit profile. In general, endpoints (Table 1) and trial design are determined collaboratively by the sponsor (for example, a pharmaceutical company) and the US Food and Drug Administration (FDA). Traditionally only one primary endpoint was used, along with several secondary endpoints. Because survival is paramount to patients, regulatory bodies such as the FDA have generally considered OS to be the major or primary endpoint/outcome of interest (FDA, 2007). However, in the past few years, there has been much debate over which endpoints should be used. Understanding the definitions of endpoints is important to the AP and the patients he or she serves. Potential endpoints are described in the paragraphs that follow.

**Table 1:**
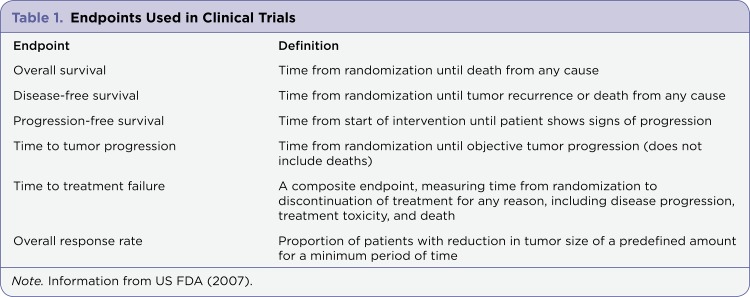
Table 1. Endpoints Used in Clinical Trials

Overall survival is considered the most desirable and reliable endpoint because it is precise and easy to measure (FDA, 2007). It is the traditional endpoint for assessing the efficacy of new treatments for metastatic breast cancer (MBC; Burzykowski et al., 2008). Overall survival, evaluated from the time of randomization until death, translates into how long a person is expected to live. However, assessing OS requires prolonged follow-up of all patients, which may ultimately result in delays in evaluating new therapies. Moreover, the potential effect on OS for first-line metastatic therapies may be diluted by the effects of subsequent therapy. Currently there is much discussion of whether other clinical endpoints or interim surrogates could be utilized initially, such as DFS, PFS, or TTP, as long as the study could be powered to eventually evaluate OS.

Disease-free survival is defined as the time from randomization until recurrence of tumor or death (from any cause; FDA, 2007). This translates into how long a person may be free of disease (i.e., no evidence of disease); it does not relate to the overall length of life. The most common use of the DFS endpoint is in the adjuvant setting after definitive therapy. Disease-free survival can also be an important primary endpoint when a large percentage of patients achieve complete response with chemotherapy or in situations where survival may be prolonged, making other survival endpoints impractical in terms of the length of time needed for follow-up.

Progression-free survival refers to the time from the start of an intervention until the patient shows signs of disease progression. Evaluation begins when the patient starts taking the drug, not at the time of randomization (as with other endpoints). Progression-free survival is often desirable because it is available earlier than OS and thereby can shorten drug development time and result in more rapid availability of efficacious therapies. Furthermore, unlike OS, PFS is not influenced by second-line treatments (Panageas et al., 2007). It is unclear whether increased PFS translates into increased OS.

Measurement of PFS is done at specific time intervals. When comparing outcomes from multiple trials, it is important to know whether the evaluation time intervals are the same, as this could potentially bias the information. For example, if one study evaluates a patient every 6 weeks and another one does so every 9 weeks, the every-9-week trial could potentially have a 3-week advantage just based on trial design (vs. actual responses). This is important when we begin to see PFS improving by a small amount of time; the question should be does this reflect what is actually occurring in the patient or is it influenced by trial design? Advanced practitioners must be knowledgeable about the designs and endpoints of each study because they are vital for translating data into treatment options and decisions. Many researchers and clinicians are now promoting PFS as a primary endpoint in MBC trials, provided the studies are powered to ultimately evaluate OS as well.

Time to tumor progression refers to the time from randomization until objective tumor progression. It does not include death. Time to tumor progression is increasingly being used as a primary endpoint for phase II and III clinical trials in MBC. Similar to PFS, TTP can also be influenced by evaluation schedules.

Time to treatment failure refers to the time from randomization to progression/failure. It is a composite endpoint reflecting the time from randomization to treatment discontinuation for any reason, including disease progression, treatment toxicity, and death. Time to treatment failure is not used as a regulatory endpoint for drug approval because it is not adequately distinguishable from other endpoints (FDA, 2007).

Overall response rate denotes the percentage of patients who experience a reduction in the size of their tumor. The amount of reduction and the specific period of time that this response must last are predetermined during the development phase of the trial. Overall response rate provides information about the likelihood of patients responding to a given treatment.

Duration of response is the length of time that a treatment response can be expected to last. This endpoint can help patients decide whether or not they wish to undergo a particular treatment. It is important to note that many of these endpoints are reported as medians, and thus there may be a wide range of individual results.

Clinical benefit has been defined in two ways: patient centered and tumor centered. *Patient-centered* clinical benefit refers to actual improvement in clinical/physical parameters such as pain, nausea, and fatigue. It can be very helpful for understanding what the patient is experiencing. The likelihood of obtaining some relief with the treatment under consideration is an important piece of information in the patient’s decision of whether to use it. However, this measure can be very subjective. For example, data are often missing from the patient’s study questionnaire form, or the form may have been completed by someone who is caring for the patient and therefore might not completely reflect the patient’s true experience. Thus, the utility of such information may be limited.

*Tumor-centered* clinical benefit focuses on the tumor response as it relates to objective tumor responses, such as complete or partial response or stable disease. The criteria for response and the length of time it must last are determined prior to initiating the study (Ohorodnyk et al., 2009).

## Selecting Endpoints in Clinical Trials

To demonstrate the issue of endpoints in clinical trials, let us look at the MBC setting as an example. The availability of many active agents makes the development of new therapies for MBC increasingly challenging and the choice of endpoints critical. A PubMed search using the terms "breast cancer–phase III" for studies published between January 2006 and December 2010 revealed the usage of various primary endpoints in MBC trials (Table 2). The studies that used OS and PFS were published more recently (2010), whereas those that used other endpoints, such as ORR, were published earlier (2006). This might suggest that, for MBC trials, investigators are moving away from ORR as a primary endpoint in favor of a stronger endpoint such as OS or PFS. However, this is speculative and based on the limited number of studies reviewed.

**Table 2:**
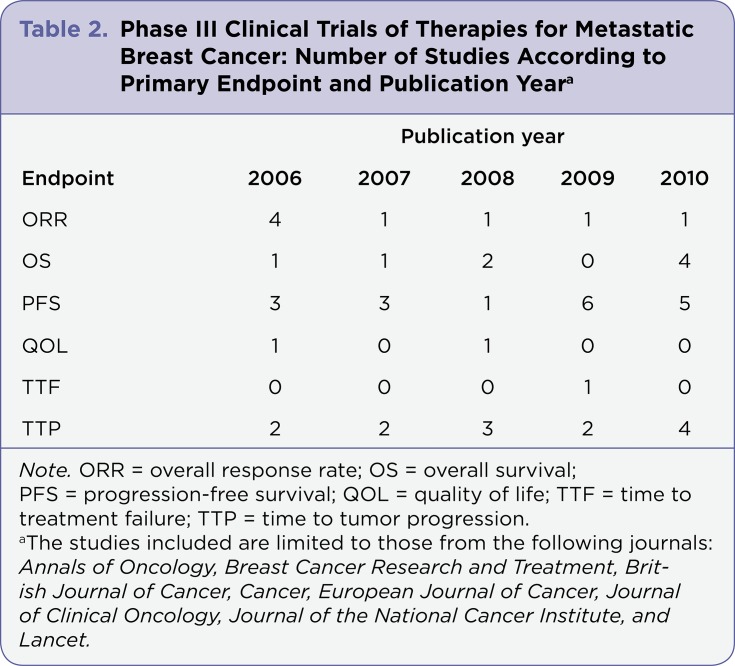
Table 2. Phase III Clinical Trials of Therapies for Metastatic Breast Cancer: Number of Studies According to Primary Endpoint and Publication Year(a)

Recent evidence suggests that many approvals of anticancer drugs have been based on endpoints other than OS (Shi & Sargent, 2009). The selection of endpoints, although collaboratively agreed upon by the sponsor and the FDA, still can vary from one study to another, which can be confusing for the clinician. Not only must you ensure that you are comparing similar endpoints, but you must also know the response evaluation time frames and the frequency of study visits, all of which can influence the ultimate outcomes.

A controversial issue surrounding PFS is whether its benefits translate into benefits in OS. Although some studies have shown that PFS correlates with OS benefits (Langley et al., 2005), other studies have not demonstrated a relationship between these two endpoints (Burzykowski et al., 2008; Miller et al., 2007). Differences in the definition of an endpoint also may complicate the decision-making process. For example, in the study by Miller et al. (2007), "progression" was defined according to the RECIST criteria (Response Evaluation Criteria in Solid Tumors), whereas Langley et al. (2005) used a combination of clinical/radiologic assessment and tumor markers. Although a meta-analysis of MBC studies indicated a positive relationship between ORR and OS based on individual patients, this relationship was not apparent when the treatment regimens were compared (Bruzzi et al., 2005). A relatively recent study also demonstrated a clear positive association between effects on progression and survival in patients with MBC, suggesting that a treatment which prolongs TTP will also result in longer survival (Sherrill et al., 2008). However, it should be noted that disease progression is subjective to measurement error and influenced by the timing of scheduled reassessments (Panageas et al., 2007).

Differences in outcomes and the definitions of primary endpoints can make treatment selection challenging. Using standard endpoint definitions may help to reduce inconsistencies and confusion when interpreting clinical trial results for daily practice.

## Secondary (Interim) Endpoints

Because the time required to assess a clinical study’s primary outcome may be lengthy, many cancer trials also examine interim or secondary outcomes such as tumor response rate (Table 1). The FDA defines ORR as the sum of partial responses and complete responses (FDA, 2007). Overall response rate is a direct measure of drug antitumor activity, which can be evaluated in a single-arm study. Symptomatic improvement is also considered a measurement of clinical benefit, especially with respect to palliation.

FDA drug approvals may use patient symptom assessments and/or physical signs representing symptomatic improvement (e.g., decrease in pain, weight loss, effusions) as secondary endpoints. However, specific measures of global health-related QOL do not serve as efficacy endpoints in oncology drug approvals. Alternatively, biomarkers may be useful to identify prognostic factors, patient selection, and stratifications for consideration. Additional research may be necessary to validate available tests and determine whether improvements in biomarkers can predict clinical benefit (FDA, 2007).

Another endpoint that may be included in clinical trials is time to a related event. Skeletal-related events (SREs), a common complication of MBC, can increase overall morbidity and mortality (Saad et al., 2007). The risk of a pathologic fracture developing within 5 years is four times greater for a patient with MBC than for a patient without MBC (Vestergaard, Rejnmark, & Mosekilde, 2009). Measuring the time to SREs is an important endpoint in MBC trials that investigate treatments to prevent skeletal adverse events. This endpoint has been used as both a primary and a secondary endpoint, depending on the type of agent(s) being examined.

## Absolute Risk vs. Relative Risk

Another important task for the AP is explaining the risks and benefits of cancer treatments to patients. The concepts of absolute risk (risk of a specific event) and relative risk (theoretical risk in comparison to another population) should be discussed with the patient if applicable (Table 3). The following vignette illustrates these concepts and the difference between them:

A patient reads or hears news about a MBC trial in which a drug has been found to reduce the risk of breast cancer, or reduce the risk of side effects or dying of breast cancer, by a certain percentage. However, the report may be misleading if the numbers represent relative risk reduction rather than absolute risk reduction. Assume that the clinical trial evaluated a new drug to prevent breast cancer and that 200 women were enrolled. Of the 100 control subjects who received a placebo pill, breast cancer developed in 2 of them. Among the 100 women in the active-treatment arm, breast cancer occurred in 1 participant. When comparing the study groups (i.e., two occurrences of breast cancer in the control group vs. one in the treatment group), the relative risk reduction in breast cancer is 50%, which may sound favorable to the patient. Therefore, people who want to avoid breast cancer might consider taking the drug despite potential side effects. But would they decide to take the drug if they knew that breast cancer would be prevented in only 1 of 100 women who took it? In this example, the absolute risk reduction was much smaller (from 2% to 1%) than the relative risk reduction.

**Table 3:**
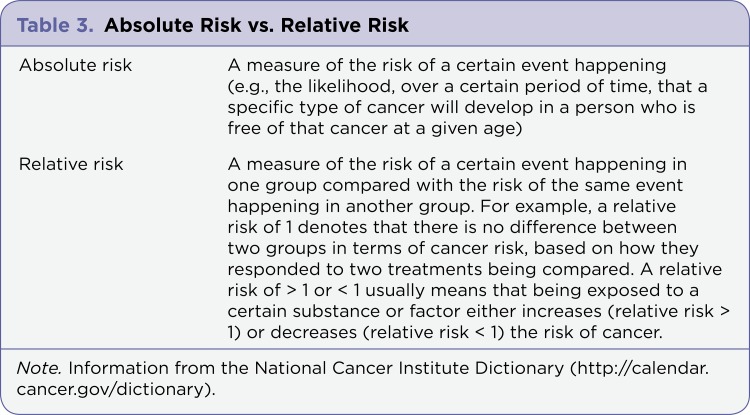
Table 3. Absolute Risk vs. Relative Risk

Relative risk is used to compare risk between two groups (e.g., drug vs. placebo), whereas absolute risk denotes the actual number of patients who obtain a specific outcome. It is very important to ensure that your patients understand these concepts and the difference between them.

## Factors That Influence Treatment Decisions: 

There is no clear method for translating a clinical trial endpoint into a "real life" clinical setting. Management decisions such as monotherapy vs. combination therapy and the optimal duration of chemotherapy should involve individualized patient assessments of disease state and symptoms, as well as clinical assessments of expected drug toxicities and potential treatment outcomes. Patients must be actively involved in discussions to evaluate their treatment preferences (Palmieri et al., 2009). Education is vital to their understanding of individual treatment options. Articulating treatment rationale to the patient is important for many reasons—psychological as well as physical.

Advanced practitioners can help patients understand *why* specific tests are ordered and *how* their results will impact treatment decisions. To optimize treatment for any type of cancer, the ability to predict how a patient will respond to a given therapy is invaluable. A recent study of patients with incurable MBC (Sheik-Yousouf, Gandhi, Dukhovny, & Verma, 2010) evaluated which endpoints and treatment benefits were believed to be the most important by patients and doctors. Many patients with MBC believed that the primary survival-related goal for a new treatment should be prolonging life by at least 1 year over the expected survival period from current best therapies. These findings contrast with the physicians’ perceptions that survival for an additional 4 to 6 months would be significant for a new treatment. This study emphasizes the need for realistic, open, and honest communication between patients and health-care providers, especially when making treatment decisions.

In addition, many patients have voiced their concern about not knowing that hospice and palliative care were also treatment options, that they were not just measures taken when no other suitable drugs or radiation therapies are available. One technique for the AP is to print out the NCCN guidelines for MBC (NCCN, 2012a; NCCN, 2012b) and write down the pros and cons of treatment options for the patient, looking at outcomes (OS, PFS, etc.), side effects, timing of therapy (weekly, monthly, etc.), and impact on overall QOL. Share this information with patients, and allow them to take the time needed to consider how the treatments might impact their life and at what cost. Decisions about treatment should not be made on the spur of the moment, but rather require thoughtful consideration. Seeing all options, including clinical trials and hospice, will help patients select the treatment that is right for them. Helping them "make sense" of the numbers will make them more comfortable with selecting their treatment.

## Conclusion

No straightforward method exists for translating data from MBC clinical trial outcomes into "real life." Trial parameters may vary, including choice of primary and secondary endpoints, the definition of endpoints, and study design. Such variability among trials can be challenging for oncologists and APs who attempt to apply study outcomes to clinical practice. Clinicians must acknowledge that endpoints evolve over time and be aware of each study design and its endpoints.

Although best evidence from clinical trials is essential to guide treatment decisions, an individual educational approach, tailored to each patient and their family, is also necessary in the decision-making process. Many patients want to participate actively in their care and treatment decisions. Individual patient histories, symptoms, preferences, and life goals must factor into treatment decisions. Treatment goals and the potential impact of treatment on the patient’s daily life must be clearly understood. Ensuring that patients have an accurate and realistic understanding of what the treatment may offer them, and how it might impact their lives and the lives of their family members, will help them make truly informed decisions. Making sure that patients are aware of all the treatment options, and giving them adequate time to consider the options and adequate time to ask questions, is part of providing quality cancer care.
